# A First-in-Human Study To Assess the Safety and Pharmacokinetics of LYS228, a Novel Intravenous Monobactam Antibiotic in Healthy Volunteers

**DOI:** 10.1128/AAC.02592-18

**Published:** 2019-06-24

**Authors:** Megan Osborn, Noah Stachulski, Haiying Sun, Johanne Blais, Vinay Venishetty, Marc Raccuglia, Martin Kankam, Richard Colvin, Florencia Segal

**Affiliations:** a Novartis Institutes for BioMedical Research, Cambridge, Massachusetts, USA; b Novartis Institutes for BioMedical Research, East Hanover, New Jersey, USA; c Novartis Institutes for BioMedical Research, Emeryville, California, USA; d Novartis Institutes for BioMedical Research, Hyderabad, India; e Novartis Institutes for BioMedical Research, Basel, Switzerland; f Vince and Associates Clinical Research, Overland Park, Kansas, USA; g Bluebird Bio, Cambridge, Massachusetts, USA

**Keywords:** *Enterobacteriaceae*, antibiotic, first-in-human study, monobactam

## Abstract

Infections caused by antibiotic-resistant Gram-negative bacteria expressing extended-spectrum β-lactamases and carbapenemases are a growing global problem resulting in increased morbidity and mortality with limited treatment options. LYS228 is a novel intravenous monobactam antibiotic targeting penicillin binding protein 3 with potent activity against Enterobacteriaceae, including multidrug-resistant clinical isolates expressing serine and metallo-β-lactamases. In this study, we evaluated the safety, tolerability, and pharmacokinetics of single and multiple intravenous doses of LYS228 in healthy volunteers. LYS228 was safe: no serious adverse events were reported. Adverse events, with the exception of catheter-related events, occurred sporadically, with similar incidences between LYS228 and placebo groups. No apparent adverse event-dose relationship was identified. LYS228 was not associated with any clinically significant dose-related hematologic, hepatic, or renal laboratory abnormalities. The most frequently observed adverse events were local injection site reactions, noted in 91.7% and 75.0% of subjects administered multiple doses of LYS228 and placebo, respectively. LYS228 demonstrated pharmacokinetic properties consistent with those of other β-lactam antibiotics, with systemic exposures slightly greater than dose proportional, short terminal half-lives (between 1.0 and 1.6 h) with no significant accumulation, and rapid clearance predominantly through urinary excretion.

## Abstract (precis)

Infections caused by antibiotic-resistant Gram-negative bacteria expressing extended-spectrum β-lactamases and carbapenemases are a growing global problem resulting in increased morbidity and mortality with limited treatment options. LYS228 is a novel intravenous monobactam antibiotic targeting penicillin binding protein 3 with potent activity against Enterobacteriaceae, including multidrug-resistant clinical isolates expressing serine and metallo-β-lactamases.

## INTRODUCTION

Infections attributed to antibiotic resistant Gram-negative bacteria represent a global threat resulting in increased patient morbidity and mortality. Infections due to Enterobacteriaceae, particularly those resistant to carbapenem antibiotics, are considered a critical priority due to the limited availability of treatment options (1, 2).

Because of their broad spectrum of antibacterial activity, carbapenems are often used for the treatment of infections caused by Gram-negative bacteria, including multidrug-resistant Enterobacteriaceae (3, 4). However, many bacterial strains have acquired the ability to express serine- and/or metallo-carbapenemases (e.g., Klebsiella pneumoniae carbapenemase and New Delhi metallo-β-lactamase 1), rendering carbapenems ineffective. These resistant strains can cause serious infections especially if left untreated. As resistant strains of Enterobacteriaceae have proliferated globally, the ability to effectively treat infections caused by these strains has been compromised, requiring the administration of poorly tolerated antibiotics, such as colistin (5, 6).

LYS228 is a monobactam antibiotic, which kills bacteria by inhibiting cell wall synthesis through covalent modification of the active-site serine of penicillin binding protein 3 (7, 8). LYS228 is active *in vitro* and *in vivo* against Enterobacteriaceae (9, 10). LYS228 is stable to most serine-β-lactamases (SBLs) and all metallo-β-lactamases (MBLs) profiled to date and has demonstrated activity against 271 Enterobacteriaceae strains, including multidrug-resistant isolates (MIC_90_, 1 μg/ml) (11). Based on MIC_90_ comparisons, LYS228 was ≥32-fold more active than aztreonam, ceftazidime, ceftazidime-avibactam, cefepime, and meropenem. Against isolates expressing extended-spectrum β-lactamases (ESBLs) (*n* = 37) and molecularly characterized KPC-producing strains (*n* = 46) and MBL-producing isolates (*n* = 33), LYS228 had MIC_90_ values of 1, 2 and 4 μg/ml, respectively (11). It is therefore expected to be effective against most bacterial infections caused by clinical strains of Enterobacteriaceae expressing single or multiple β-lactamases. Monocyclic β-lactams, like the monobactams, retain activity against MBL-expressing Enterobacteriaceae; however, MBLs are frequently coexpressed with SBLs, many of which can inactivate the clinically used monobactam aztreonam. The prevalence of strains expressing New Delhi metallo-β-lactamase 1 (NDM-1) is as high as 12% in clinical isolates from patients with invasive infections in India (12, 13), and infections caused by strains expressing NDM-1 and other MBLs have been detected globally (14). Further, the prevalence of MBL-expressing Enterobacteriaceae is increasing in environmental isolates and is expected to increase rapidly in the clinic, as they are likely to be selected following the recent introduction of novel β-lactam antibiotics, such as ceftazidime-avibactam, ceftolozane-tazobactam, and meropenem-vaborbactam, that treat strains expressing SBLs but not MBLs.

Plasma protein binding of LYS228 was low, ranging from 8.3 to 17.5% in humans. From nonclinical pharmacokinetic (PK) studies, metabolism of LYS228 was qualitatively similar in rat, dog, and human hepatocytes. LYS228 underwent β-lactam hydrolysis (M2), N-desulfonation (M1), and the combination of these reactions (M3). Across the different preclinical species, LYS228 was primarily eliminated through the renal route, with a short half-life (*t*_1/2_; 0.3 to 0.75 h). The objectives of this first-in-human study were to evaluate the safety, tolerability, and pharmacokinetics of single and multiple doses of LYS228 in healthy volunteers.

## RESULTS

### Subject demographics.

Overall, 80 subjects were enrolled and 70 completed the study. For the single ascending-dose (SAD) part, 40 subjects were enrolled and all subjects (100%) completed the study. For the multiple ascending-dose (MAD) part, 40 subjects were enrolled, of whom 30 subjects (75%) completed the study. In cohort 2 (3,000 mg) of the MAD, five subjects discontinued the study, and the cohort was repeated (cohort 2b). In cohorts 2b and 3, 24 subjects were randomized and 5 were discontinued from the study. The primary reasons for study discontinuation were adverse events in 8 subjects (4 who received 3,000 mg of LYS228 in cohort 2, 2 who received 3,000 mg of LYS228 in cohort 2b, and 2 in the pooled placebo group), personal reasons (1 subject who received 3,000 mg of LYS228 in cohort 2), and being lost to follow-up (1 subject who received 4,000 mg of LYS228 in cohort 3). The demographic data are shown in Table 1 (SAD) and Table 2 (MAD).

**TABLE 1:** Subject demographics for the SAD group

Parameter	Type of value	Value for indicated treatment					
		LYS228, *n* = 6					Placebo, *n* = 10
		300 mg	1,000 mg	2,000 mg	3,000 mg	6,000 mg	
Age (yrs)	Mean (SD)	38.5 (11.74)	44.0 (15.91)	42.5 (10.11)	42.0 (12.17)	39.7 (15.16)	38.5 (12.14)
	Median	34.5	44.5	40.0	43.5	36.0	34.5
	Range	27, 58	26, 60	30, 57	25, 59	25, 60	24, 56
Sex, no. (%)	Male	6 (100)	4 (66.7)	5 (83.3)	6 (100)	5 (83.3)	9 (90.0)
	Female	0	2 (33.3)	1 (16.7)	0	1 (16.7)	1 (10.0)
Predominant race, no. (%)	Black or African American	5 (83.3)	3 (50.0)	4 (66.7)	2 (33.3)	3 (50.0)	7 (70.0)
	White	1 (16.7)	3 (50.0)	2 (33.3)	4 (66.7)	3 (50.0)	3 (30.0)
Ethnicity, no. (%)	Hispanic or Latino	0	0	0	2 (33.3)	1 (16.7)	0
	Not Hispanic or Latino	6 (100)	6 (100)	6 (100)	4 (66.7)	5 (83.3)	10 (100)
Wt (kg)	Mean (SD)	86.95 (15.970)	78.07 (12.246)	83.70 (4.053)	84.68 (8.823)	86.32 (13.413)	85.24 (12.098)
	Median	84.60	78.25	82.05	83.35	84.35	84.45
	Range	63.8, 110.2	62.0, 97.5	80.0, 89.9	72.8, 95.1	69.4, 101.5	60.7, 108.7
Ht (cm)	Mean (SD)	179.00 (5.215)	173.75 (8.478)	173.17 (7.394)	174.92 (5.713)	175.58 (8.096)	175.45 (7.492)
	Median	177.50	173.25	171.25	176.50	177.75	178.00
	Range	174.0, 188.0	162.5, 188.0	165.0, 184.0	165.5, 181.0	160.0, 182.5	161.0, 186.0
Body mass index (kg/m^2^)	Mean (SD)	27.02 (3.847)	25.78 (2.753)	28.02 (2.114)	27.72 (2.881)	27.97 (3.656)	27.73 (3.585)
	Median	27.45	26.50	28.40	28.45	27.05	28.45
	Range	20.1, 31.2	21.1, 28.8	24.5, 30.3	23.2, 31.0	24.2, 33.1	18.9, 31.4

**TABLE 2:** Subject demographics for the MAD group

Parameter	Type of value	Value for indicated treatment				
		LYS228, *n* = 6				Placebo q8h, *n* = 16
		2,000 mg q8h^*a*^	3,000 mg q8h	3,000 mg^*b*^ q8h	4,000 mg q8h	
Age (yrs)	Mean (SD)	42.8 (15.16)	43.5 (10.73)	40.7 (17.24)	34.0 (9.38)	33.8 (9.66)
	Median	47.0	42.0	43.0	31.5	30.0
	Range	24, 58	32, 58	21, 57	23, 50	24, 52
Sex, no. (%)	Male	6 (100)	2 (33.3)	6 (100)	6 (100)	13 (81.3)
	Female	0	4 (66.7)	0	0	3 (18.8)
Predominant race, no. (%)	Black or African American	3 (50.0)	2 (33.3)	2 (33.3)	4 (66.7)	10 (62.5)
	Native Hawaiian or Other Pacific Islander	0	0	0	1 (16.7)	0
	White	3 (50.0)	4 (66.7)	4 (66.7)	1 (16.7)	6 (37.5)
Ethnicity, no. (%)	Hispanic or Latino	1 (16.7)	0	0	0	0
	Not Hispanic or Latino	5 (83.3)	6 (100)	6 (100)	6 (100)	16 (100)
Wt (kg)	Mean (SD)	76.97 (12.655)	69.22 (10.470)	85.63 (17.122)	86.83 (9.836)	82.98 (11.202)
	Median	75.50	70.15	85.65	84.50	81.00
	Range	62.1, 97.5 52.4	82.8 66.1	105.1 75.4	101.1 60.4	101.7
Ht (cm)	Mean (SD)	169.25 (8.739)	163.17 (5.125)	180.75 (1.891)	175.00 (8.396)	173.75 (7.685)
	Median	170.00	163.75	180.50	173.75	173.50
	Range	156.0, 180.5	156.0, 168.5	178.0, 183.5	165.0, 190.0	160.0, 186.5
Body mass index (kg/m^2^)	Mean (SD)	26.92 (4.067)	26.17 (4.926)	26.15 (4.809)	28.42 (3.190)	27.54 (3.656)
	Median	28.90	26.10	26.30	28.65	29.55
	Range	20.3, 30.0	18.5, 34.0	20.9, 31.7	24.2, 32.5	20.8, 31.6

a q8h, every 8 h.

b Repeated cohort (2b).

### Safety and tolerability.

There were no deaths or serious adverse events during the study. In the SAD group, no subject discontinued due to an adverse event. Six of 40 (15%) subjects experienced seven adverse events. The incidence of adverse events was generally low: all were assessed as grade 1 in severity, and no clear dose-related trends were observed. The incidence of adverse events by treatment group is provided in Table 3. One subject who received 3,000 mg of LYS228 experienced 2 adverse events, injection site pain and peripheral swelling, with both suspected to be related to the study drug. In the MAD group, 9 subjects discontinued the study drug due to an adverse event, with 8 also discontinuing from the study. Thirty-six of 40 (90%) subjects experienced 101 adverse events (Table 4). The most common adverse events reported were infusion site phlebitis (*n* = 26 [65.0%]), infusion site extravasation (*n* = 10 [25.0%]), headache (*n* = 6 [15.0%]), infusion site pain (*n* = 4 [10.0%]), and presyncope (*n* = 3 [7.5%]). Infusion site phlebitis was more frequent in subjects administered LYS228 (21/24 [87.5%]) than in those given placebo (5/16 [31.3%]). Because of the multiple terms used to describe catheter-related adverse events (Table 5), an analysis was done to determine the number of subjects with at least one of these events. All subjects that received placebo or LYS228 (2,000 mg and 3,000 mg) in cohorts 1 and 2 experienced catheter-related adverse events. Thirty-five subjects (87.5%) who received either LYS228 (22/24 [91.7%]) or placebo (13/16 [81.3%]) had adverse events that were suspected to be related to the study drug. The most frequent events suspected to be related to the study drug were phlebitis, injection site pain, peripheral swelling, infusion site swelling, infusion site hemorrhage, infusion site reaction, infusion site edema, and infusion site extravasation. All adverse events were reported to be grade 1 (49 [70%]) or grade 2 (52 [67.5%]), with most of the grade 2 events consisting of phlebitis or other catheter-related events. Although CTCAE v4.03 requires that infusion site phlebitis be assigned grade 2 severity, the investigator assessed all of these events as clinically mild.

**TABLE 3:** Incidence of adverse events in SAD group

AE^*a*^	No. (%) for indicated treatment					
	LYS228, *n* = 6					Placebo, *n* = 10
	300 mg	1,000 mg	2,000 mg	3,000 mg	6,000 mg	
Subjects with at least one AE	1 (16.7)	1 (16.7)	1 (16.7)	2 (33.3)	0	1 (10.0)
Congestion, nasal	1 (16.7)	0	0	1 (16.7)	0	0
Infusion site pain	0	1 (16.7)	0	0	0	0
Injection site pain	0	0	0	1 (16.7)	0	0
Nightmare	0	0	0	0	0	1 (10.0)
Peripheral swelling	0	0	0	1 (16.7)	0	0
Skin discoloration	0	0	1 (16.7)	0	0	0

a Arranged in descending order of frequency (in total group) and alphabetically by preferred term. AE, adverse event.

**TABLE 4:** Incidence of adverse events in MAD group

AE^*a*^	No. (%) for indicated treatment				
	LYS228, *n* = 6				Placebo q8h, *n* = 16
	2,000 mg q8h	3,000 mg q8h	3,000 mg^*b*^ q8h	4,000 mg q8h	
Subjects with at least one AE	6 (100)	6 (100)	5 (83.3)	5 (83.3)	14 (87.5)
Infusion site phlebitis	6 (100)	6 (100)	5 (83.3)	4 (66.7)	5 (31.3)
Infusion site extravasation	0	1 (16.7)	2 (33.3)	2 (33.3)	5 (31.3)
Headache	1 (16.7)	3 (50.0)	0	1 (16.7)	1 (6.3)
Infusion site pain	0	0	0	0	4 (25.0)
Presyncope	1 (16.7)	0	1 (16.7)	1 (16.7)	0
Dizziness	0	1 (16.7)	0	0	1 (6.3)
Infusion site haemorrhage	1 (16.7)	0	0	0	1 (6.3)
Infusion site inflammation	0	0	0	2 (33.3)	0
Injection site phlebitis	0	0	0	1 (16.7)	1 (6.3)
Abdominal pain	0	0	0	0	1 (6.3)
Abdominal pain, upper	0	0	0	0	1 (6.3)
Dermatitis, contact	1 (16.7)	0	0	0	0
Epistaxis	0	0	1 (16.7)	0	0
Fatigue	0	0	0	0	1 (6.3)
Feeling hot	0	0	0	0	1 (6.3)
Infusion site erythema	0	1 (16.7)	0	0	0
Infusion site edema	0	1 (16.7)	0	0	0
Infusion site swelling	1 (16.7)	0	0	0	0
Nausea	0	1 (16.7)	0	0	0
Rash	0	0	0	0	1 (6.3)
Thrombophlebitis, superficial	0	0	1 (16.7)	0	0
Tinnitus	0	0	0	0	1 (6.3)
Upper respiratory tract infection	0	0	0	0	1 (6.3)
Vessel puncture site haematoma	1 (16.7)	0	0	0	0
Vessel puncture site hemorrhage	0	1 (16.7)	0	0	0

a Arranged in descending order of frequency (in total group) and alphabetically by preferred term.

b Repeated cohort.

**TABLE 5:** Incidence of catheter-related adverse events in MAD group

AE^*a*^	No. (%) for indicated treatment					
	LYS228, *n* = 6					Placebo q8h, *n* = 16
	False head	2,000 mg q8h	3,000 mg q8h	3,000 mg^*b*^ q8h	4,000 mg q8h	
Subjects with at least one i.v. site-related AE	6 (100)	6 (100)	5 (83.3)	5 (83.3)	22 (91.7)	12 (75.0)
Infusion site phlebitis	6 (100)	6 (100)	5 (83.3)	4 (66.7)	21 (87.5)	5 (31.3)
Infusion site extravasation	0	1 (16.7)	2 (33.3)	2 (33.3)	5 (20.8)	5 (31.3)
Infusion site pain	0	0	0	0	0	4 (25.0)
Infusion site hemorrhage	1 (16.7)	0	0	0	1 (4.2)	1 (6.3)
Infusion site inflammation	0	0	0	2 (33.3)	2 (8.3)	0
Injection site phlebitis	0	0	0	1 (16.7)	1 (4.2)	1 (6.3)
Infusion site erythema	0	1 (16.7)	0	0	1 (4.2)	0
Infusion site edema	0	1 (16.7)	0	0	1 (4.2)	0
Infusion site swelling	1 (16.7)	0	0	0	1 (4.2)	0
Vessel puncture site hematoma	1 (16.7)	0	0	0	1 (4.2)	0
Vessel puncture site hemorrhage	0	1 (16.7)	0	0	1 (4.2)	0

a Arranged in descending order of frequency (in total group) and alphabetically by preferred term. i.v., intravenous.

b Repeated cohort.

Because of the high rate of catheter-related adverse events in all subjects, the procedures related to placement and maintenance of intravenous catheters were reviewed and changes in how the catheters were placed and maintained were implemented. These changes resulted in fewer catheter-related events in cohorts 2b and 3a, during which 10 of 12 subjects who received LYS228 (83.3%) and 9 of 12 subjects who received placebo (75%) had catheter-related events. In addition, 66.7% of subjects administered LYS228 prior to the changes discontinued the study drug due to one of these catheter-related events, compared with 33.3% after the changes.

A number of parameters were evaluated to further determine if there were significant differences in catheter-related events in subjects who received LYS228 or placebo. The average times from placement of a catheter until its removal for subjects who received LYS228 and placebo were 37.4 and 50.2 h, respectively. In addition, the mean number of catheters per subject was 3.3 for those who received LYS228 and 2.6 for those who received placebo. However, the mean rates of catheter-related events per catheter were similar for subjects who received LYS228 (0.7) and placebo (0.6).

In the SAD and MAD groups, there were no clinically significant changes to vital signs, electrocardiogram (ECG), urinalysis, hematology, or chemistry parameters tested.

### Pharmacokinetics.

**FIG 1:**
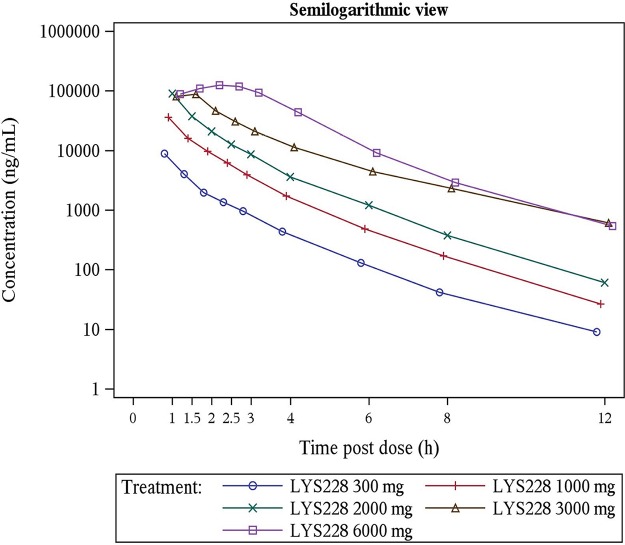
Arithmetic mean (SD) plasma concentration-time profiles for the SAD group.

Fifty-four subjects were included in the pharmacokinetics analysis set. In the SAD group, following single intravenous infusions, the median time to maximum concentration of drug (*T*_max_) of LYS228 in plasma ranged from 1 to 2.25 h. Mean maximum concentration of drug (*C*_max_) (in the range of 8.82 to 129.0 μg/ml) and last area under the concentration-time curve (AUC_last_) (in the range of 12.2 to 416.0 μg·h/ml) increased with increasing dose over the 300- to 6,000-mg dose range (Table 6). A statistically significant deviation from dose proportionality for AUC_last_ (slope, 1.17; 90% confidence interval [CI], 1.12 to 1.23) and AUC to infinity (AUC_inf_) (slope, 1.18, 90% CI, 1.12 to 1.23) was observed over the dose range of 300 to 6,000 mg, and none of the primary pharmacokinetic parameters can be considered proportional to dose. In fact, for a 20-fold increase in dose (300 to 6,000 mg), AUC_last_ increased by a factor of 33.7 and AUC_inf_ increased by a factor of 33.9, indicating an increase in AUC that was more than dose proportional. Dose proportionality was not relevant to *C*_max_, as infusion durations across the cohorts were different. After the end of the infusion, plasma concentrations decreased rapidly, with mean elimination half-lives ranging from 1.29 to 1.60 h. The arithmetic mean plasma concentration-time profiles following single ascending doses are presented in Fig. 1. Percent time over MIC (%*T*_>MIC_) was calculated using a target MIC of 1 μg/ml, corresponding to the MIC_90_ value for combined results from survey studies of LYS228 against clinical isolates of Enterobacteriaceae (*n* = 1,327; composed largely of multidrug-resistant strains [data not shown]). Mean %*T*_>MIC_ values increased with dose (21.1, 38.9, 50.3, 75.9, and 87.7 for 300-, 1,000-, 2,000-, 3,000-, and 6,000-mg doses, respectively) (Table 6). The mean percentage of LYS228 recovered from the urine samples collected up to 8 h and 72 h ranged from 70.8 to 78.7%. Urinary excretion of LYS228 was essentially complete by 8 h, independent of dose, and less than 5% of the dose was recovered during the 8-h to 72-h time period. The calculated mean renal clearance (CL_R_) ranged from 11.5 to 18.6 liters/h (0.14 to 0.21 liter/h/kg), suggesting a major renal elimination route for LYS228 given that the systemic clearance ranged from 15.6 to 25.2 liters/h (0.18 to 0.29 liter/h/kg).

**TABLE 6:** Summary statistics of PK parameter values for SAD group^*a*^

PK parameter	Value for LYS228 at indicated dose, *n* = 6				
	300 mg	1,000 mg	2,000 mg	3,000 mg	6,000 mg
*C*_max_ (μg/ml)	8.82 ± 1.24 (14.1) [6]	35.80 ± 7.340 (20.5) [6]	89.7 ± 16.9 (18.9) [6]	87.0 ± 26.5 (30.5) [5]	129.0 ± 34.3 (26.6) [6]
*T*_max_ (h)	1.01 (1.00–1.02) [6]	1.00 (1.00–1.07) [6]	1.00 (1.00–1.02) [6]	1.50 (1.00–2.50) [5]	2.25 (2.00–2.63) [6]
AUC_last_ (μg·h/ml)	12.20 ± 2.56 (20.9) [6]	49.80 ± 3.48 (7.0) [6]	118.0 ± 23.2 (19.6) [6]	186.0 ± 18.9 (10.2) [5]	416.0 ± 143.0 (34.4) [6]
AUC_inf_ (μg·h/ml)	12.30 ± 2.57 (20.9) [6]	49.90 ± 3.48 (7.0) [6]	118.0 ± 23.2 (19.6) [6]	189.0 ± 20.5 (10.9) [5]	417.0 ± 143.0 (34.4) [6]
AUC_0–8_ (μg·h/ml)	12.1 ± 2.5 (20.6) [6]	49.40 ± 3.52 (7.1) [6]	117.0 ± 23.2 (19.8) [6]	180.0 ± 18.6 (10.4) [5]	409.0 ± 141.0 (34.5) [6]
*t*_1/2_ (h)	1.41 ± 0.124 (8.8) [6]	1.40 ± 0.173 (12.4) [6]	1.36 ± 0.112 (8.2) [6]	1.60 ± 0.525 (32.9) [5]	1.29 ± 0.148 (11.5) [6]
*V*_ss_ (ml)	30,800 ± 4,310 (14.0) [6]	25,300 ± 5,180 (20.4) [6]	20,900 ± 4,730 (22.6) [6]	27,700 ± 18,500 (66.6) [5]	18,700 ± 4,310 (23.0) [6]
*V*_ss_ (ml/kg)	354.2 ± 49.6 (14.0) [6]	324.1 ± 66.4 (20.4) [6]	249.7 ± 56.5 (22.6) [6]	327.1 ± 218.5 (66.6) [5]	216.6 ± 49.9 (23.0) [6]
CL (ml/h)	25,200 ± 4460 (17.7) [6]	20,100 ± 1,420 (7.1) [6]	17,500 ± 3,670 (21.0) [6]	16,000 ± 1,730 (10.8) [5]	15,600 ± 4,210 (27.1) [6]
CL (mL/h/kg)	289.8 ± 51.3 (17.7) [6]	257.5 ± 18.2 (7.1) [6]	209.1 ± 43.8 (21.0) [6]	188.9 ± 20.4 (10.8) [5]	180.7 ± 48.8 (27.1) [6]
%*T*_>MIC_ at 1 ug/ml	21.1 ± 3.72 (17.7) [6]	38.9 ± 4.91 (12.6) [6]	50.3 ± 2.72 (5.4) [6]	75.9 ± 30.2 (39.8) [5]	87.7 ± 10.5 (12.0) [6]

a Statistics are means ± SDs (coefficient of variation [percent]) [number]. The coefficient of variation is determined as follows: SD/mean × 100. For *T*_max_, statistics are median (minimum to maximum) [number]. Statistics are for day 1.

**FIG 2:**
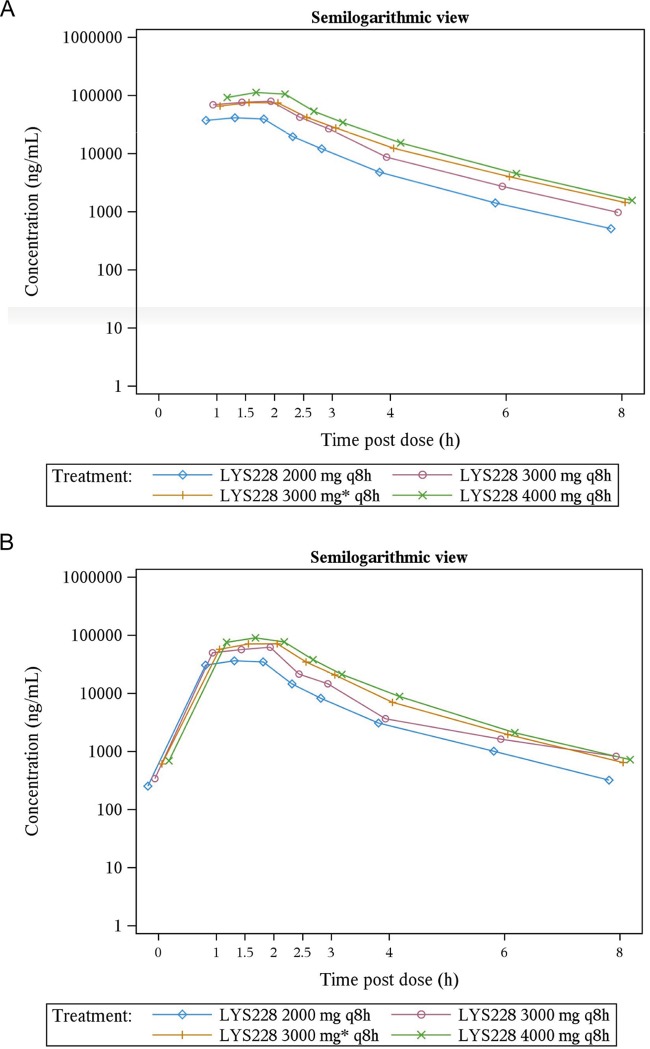
Arithmetic mean (SD) plasma concentration-time profiles for the MAD group.

In the MAD group, following multiple intravenous infusions, plasma concentrations of LYS228 reached maximum at median *T*_max_s ranging from 1.51 to 1.77 h on day 1 and 1.5 to 2 h on day 6 over the dose range of 2,000 to 4,000 mg every 8 h. Mean *C*_max_ and AUC from 0 to 8 h (AUC_0–8_) on day 1 were in the range of 41.8 to 114.0 μg/ml and 97.3 to 262.0 μg h/ml, respectively. The corresponding mean *C*_max_ and AUC_0–8_ values on day 6 were 36.2 to 86.1 μg/ml and 78.5 to 184.0 μg·h/ml, respectively (Table 7). Trends toward a decrease in mean AUC and an increase in mean clearance were observed on day 6 compared to day 1 across the doses, but the significance was not evaluated statistically. There was a statistically significant deviation from dose proportionality for *C*_max_, AUC_last_, and AUC_inf_ over the dose range of 2,000 to 4,000 mg on day 1 and day 6, and none of these primary pharmacokinetic parameters can be considered proportional to dose. Similar to the case with the SAD group, a 2-fold increase in dose (2,000 mg to 4,000 mg) resulted in a 2.5-fold increase in *C*_max_ and 2.4-fold increases in AUCs after first dose administration on day 6, indicating an increase in exposure that was more than dose proportional. The elimination half-life was in the range of 1.10 to 1.17 h over the 2,000- to 4,000-mg dose range on day 1, and it was 1.04 to 1.22 h on day 6. The mean accumulation index (*R*_acc_) was determined to be in the range of 0.74 to 0.81 and indicated no accumulation of LYS228 in systemic circulation. The arithmetic mean plasma concentration-time profiles for multiple ascending doses on days 1 and 6 are presented in Fig. 2. Percent time over MIC showed no significant differences in these values across the cohorts on day 1 and day 6. Mean %*T*_>MIC_ values were determined to be 80.6, 100, and 103 for doses of 2,000, 3,000, and 4,000 mg every 8 h, respectively, on day 1 and 72.7, 83.5, and 87.1 for doses of 2,000, 3,000, and 4,000 mg every 8 h, respectively, on day 6. Mean %*T*_>MIC_ values in the MAD were higher than the SAD, indicating that with multiple doses, concentrations were above MIC for longer periods of time than with a single dose. The mean percentages of LYS228 recovered from the urine collected over 8-h intervals were 87.7% and 68.8% on day 1 and 77.6 to 98.3% on day 6 over a dose range of 2,000 to 4,000 mg every 8 h. The mean systemic clearance ranged from 15.8 to 21.5 liters/h (0.18 to 0.28 liter/h/kg) and 20.4 to 26.5 liters/h (0.24 to 0.34 liter/h/kg) on day 1 and day 6, respectively, across all MAD cohorts.

**TABLE 7:** Summary statistics of PK parameter values for MAD group^*a*^

PK parameter	Profile day	Value for LYS228 at indicated dose (*n* = 6)			
		2,000 mg q8h	3,000 mg q8h	3,000 mg^*b*^ q8h	4,000 mg q8h
*C*_max_ (μg/ml)	1	41.8 ± 11.0 (26.3) [6]	79.90 ± 8.84 (11.1) [6]	77.2 ± 19.4 (25.2) [6]	114.0 ± 20.2 (17.7) [6]
	6	36.20 ± 9.17 (25.3) [6]	61.8 [1]	74.2 ± 23.1 (31.1) [4]	86.1 ± 15.5 (18.0) [5]
*T*_max_ (h)	1	1.51 (1.50–2.02) [6]	1.77 (1.50–2.02) [6]	1.75 (1.50–2.03) [6]	1.50 (1.50–2.00) [6]
	6	1.50 (1.50–2.00) [6]	2.00 (2.00–2.00) [1]	1.81 (1.55–2.00) [4]	1.50 (1.50–2.00) [5]
AUC_last_ (μg·h/ml)	1	97.3 ± 24.5 (25.2) [6]	188.0 ± 29.4 (15.6) [6]	194.0 ± 59.80 (30.7) [6]	262.0 ± 50.5 (19.3) [6]
	6	78.5 ± 18.5 (23.5) [6]	127.0 [1]	161.0 ± 59.2 (36.8) [4]	184.0 ± 28.7 (15.6) [5]
AUC_inf_ (μg·h/ml)	1	98.1 ± 25.0 (25.4) [6]	190.0 ± 29.8 (15.7) [6]	197.0 ± 60.9 (30.9) [6]	264.0 ± 51.3 (19.4) [6]
	6	79.1 ± 18.6 (23.5) [6]	130.0 [1]	162.0 ± 59.9 (37.0) [4]	185.0 ± 29.0 (15.7) [5]
AUC_0–8_ (μg·h/ml)	1	97.3 ± 24.5 (25.2) [6]	188.0 ± 29.4 (15.6) [6]	195.0 ± 59.8 (30.8) [6]	262.0 ± 50.5 (19.3) [6]
	6	78.5 ± 18.5 (23.5) [6]	127.0 [1]	161.0 ± 59.2 (36.8) [4]	184.0 ± 28.7 (15.6) [5]
*t*_1/2_ (h)	1	1.11 ± 0.0943 (8.5) [6]	1.10 ± 0.182 (16.6) [6]	1.13 ± 0.126 (11.1) [6]	1.17 ± 0.129 (11.0) [6]
	6	1.22 ± 0.222 (18.2) [6]	1.86 [1]	1.17 ± 0.0323 (2.8) [4]	1.04 ± 0.138 (13.3) [5]
*V*_ss_ (ml)	1	23,500 ± 4,210 (17.9) [6]	18,100 ± 1,870 (10.4) [6]	21,100 ± 4,020 (19.0) [6]	18,600 ± 2,800 (15.0) [6]
	6	26,800 ± 4,980 (18.6) [6]	25,600 [1]	21,100 ± 5,510 (26.1) [4]	22,000 ± 3,850 (17.5) [5]
*V*_ss_ (ml/kg)	1	305.3 ± 54.7 (17.9) [6]	261.5 ± 27.0 (10.4) [6]	246.4 ± 46.9 (19.0) [6]	214.2 ± 32.2 (15.0) [6]
	6	348.2 ± 64.7 (18.6) [6]	369.8 [1]	246.4 ± 64.3 (26.1) [4]	253.4 ± 44.3 (17.5) [5]
CL (ml/h)	1	21,500 ± 4,440 (20.7) [6]	16,200 ± 2,370 (14.6) [6]	16,400 ± 3,880 (23.7) [6]	15,700 ± 2,960 (18.8) [6]
	6	26,500 ± 5,330 (20.1) [6]	23,500 [1]	20,400 ± 6,650 (32.5) [4]	22,200 ± 3,380 (15.2) [5]
CL (ml/h/kg)	1	279.3 ± 57.7 (20.7) [6]	234.0 ± 34.2 (14.6) [6]	191.5 ± 45.3 (23.7) [6]	180.8 ± 34.1 (18.8) [6]
	6	344.3 ± 69.2 (20.1) [6]	339.5 [1]	238.2 ± 77.7 (32.5) [4]	255.7 ± 38.9 (15.2) [5]
%*T*_>MIC_ at 1 μg/ml	1	80.6 ± 7.32 (9.1) [6]	92.4 ± 7.06 (7.6) [6]	100 ± 10.7 (10.6) [6]	103 ± 9.20 (8.9) [6]
	6	72.7 ± 7.20 (9.9) [6]	87.5 [1]	83.5 ± 11.6 (13.9) [4]	87.1 ± 12.2 (14.0) [5]

a Statistics are means ± SDs (coefficient of variation [percent]) [number]. The coefficient of variation is determined as follows: SD/mean × 100. For *T*_max_, statistics are median (minimum to maximum) [number].

b Repeated cohort.

## DISCUSSION

LYS228 was safe when administered intravenously as single and multiple doses to healthy subjects. There were no serious adverse events, and with the exception of infusion site phlebitis, the rates of adverse events were similar between LYS228- and placebo-treated subjects. The most frequently observed adverse events were catheter or infusion related, and one or more of these events occurred in 91.7% and 75% of subjects who received multiple doses of LYS228 or normal saline, respectively. These catheter- or infusion-related events occurred with similar frequencies among subjects receiving LYS228 and placebo, with the exception of infusion site phlebitis, which was noted in 87.5% and 31.3% of subjects administered multiple doses of LYS228 and placebo, respectively.

In this study, LYS228 and placebo administration resulted in high rates of local infusion reactions and phlebitis. Improved catheter-related procedures implemented during the study resulted in a slight decline in the overall rate of such events. These rates are consistent with the rates of phlebitis reported in randomized studies of hospitalized patients; in those studies, phlebitis was associated with 42% of catheters among 1,054 catheters, and patients that received antibiotics had a higher (2 times) relative risk of phlebitis than did all other indications for intravenous therapy (15). The results in this study are also consistent with a recent first-in-human study for vaborbactam in which the rate of phlebitis among subjects who received placebo was 50% (16). In the subsequent phase 3 studies, the rate dropped, and the rates in the prescribing information are 4.4% for meropenem-vaborbactam and 0.7% for piperacillin-tazobactam (17). Local reactions, such as phlebitis or thrombophlebitis following intravenous administration of aztreonam to patients, occur at rates of 1.9% to 2.4%, but the rate may be higher depending on how they are assessed (18, 19). Local reactions should be closely monitored in future LYS228 studies in patients. Should there be dose-limiting phlebitis in future LYS228 studies, additional mitigation strategies, such as a change in the LYS228 diluent and/or changes in the infusion duration, may be required.

The PK of LYS228 was consistent with those of other beta-lactam antibiotics, such as meropenem. Systemic exposure of LYS228 was slightly greater than dose proportional, the half-life was between 1.0 and 1.6 h (with no significant accumulation), and clearance was rapid and predominantly through urinary excretion (20–22).

LYS228 demonstrated efficacy *in vivo* in a neutropenic murine thigh infection model against E. coli and K. pneumoniae strains, including strains expressing KPC and NDM carbapenemases (9, 10). Using this model, it was determined that the predominant PK/pharmacodynamic (PD) parameter associated with antibacterial efficacy of LYS228 was the percentage of the dosing interval that free drug concentrations remained above the MIC of the infecting organism (%*T*_>MIC_) (9, 10).

The results of this study indicate that LYS228 has a favorable safety, tolerability, and PK profile. These data suggest that LYS228 can be used in patients with infections caused by Enterobacteriaceae in future phase 2 clinical trials.

## MATERIALS AND METHODS

### Study design.

This was a randomized, placebo-controlled study of single and multiple ascending intravenous doses in healthy volunteers. The study was conducted at a single site in the United States. The study protocol, amendments, and informed consent were reviewed and approved by an Independent Review Board and executed in accordance with the Declaration of Helsinki and International Conference on Harmonisation and Good Clinical Practice. All subjects provided written informed consent before any study-related procedure took place.

The study was divided into two sequential parts. The single ascending dose (SAD) part consisted of five sequential cohorts of eight healthy subjects randomized in a 3:1 ratio to LYS228 or matching placebo (300, 1,000, 2,000, 3,000, and 6,000 mg [cohorts 1, 2, 3, 4, and 5, respectively]). Subjects received a single intravenous dose of LYS228 or placebo with a 1-h infusion duration for cohorts 1, 2, and 3, a 1.5-h infusion duration for cohort 4, and a 3-h infusion duration for cohort 5. The multiple ascending dose (MAD) part consisted of four cohorts in which subjects were randomized to receive LYS228 or matching placebo (2,000, 3,000, 3,000, and 4,000 mg) every 8 h infused over 2 h for 5 days. In the first two MAD cohorts, eight healthy subjects were randomized in a 3:1 ratio to LYS228 or matching placebo. Subsequently, in the second 3,000-mg cohort and the 4,000-mg cohort, 12 subjects were randomized in a 1:1 ratio to LYS228 or placebo. All subjects and the investigator and study nurse were blinded to treatment assignment. Designated sponsor staff were unblinded from the study start (PK sample analyst and study statistician) or allowed to receive unblinded results (clinical trial team) as required for safety and decision-making during dose escalation.

The first MAD cohort was initiated only after the interim analysis of safety and PK data from the SAD had been completed. The sponsor’s clinical team and the investigator prior to dose escalation decisions reviewed safety data jointly.

### Subjects.

Healthy male and female (of non-childbearing potential) subjects of 18 to 60 years of age were eligible. Subjects had to weigh at least 50 kg and have a body mass index (BMI) between 18 and 36 kg/m^2^. Exclusion criteria included history of hypersensitivity to similar antibiotic drug classes (penicillin, cephalosporin, carbapenem, or monobactam), history of autonomic dysfunction, immunodeficiency diseases, chronic hepatitis, seizures, significant gastrointestinal illness, antibiotic-associated diarrhea within 1 year, and clinically significant electrocardiogram (ECG) and laboratory abnormalities. Subjects were also excluded if they had impaired renal function (creatinine clearance < 80 ml/min), urinary obstruction, or difficulty voiding or had used any prescription drugs or herbal supplements within 4 weeks of dosing or over-the-counter medication or dietary supplements within 2 weeks of dosing.

### Safety assessments.

Safety assessments consisted of collecting information regarding all adverse events, with their severity and relationship to study drug, physical examinations, and laboratory evaluations.

### Bioanalytical methods.

Concentrations of LYS228 in plasma and urine were determined by a validated liquid chromatography-tandem mass spectrometry (LC-MS/MS) method, described below.

**(i) Analysis of LYS282 in plasma samples.** LYS228 was determined in human plasma via a validated analytical method, consisting of protein precipitation followed by reverse-phase liquid chromatography with tandem mass spectrometric detection. The linearity of the analytical method for analysis of LYS228 was validated (linear regression) in the range of 2 (lower limit of quantification [LLOQ]) to 2,000 ng/ml using 50 μl of plasma. The method was specific in human plasma (maximum interference, 9.65% of signal at LLOQ in blank plasma and 0.07% in zero sample). The interday accuracy and precision of the method were evaluated as the mean bias and precision of quality control (QC) samples analyzed during 3 validation days: bias at LLOQ was −9.5%, and precision was 8.12%. Above LLOQ, the biases were within the range of −0.33% to 0.17% and the precisions were within the range 4.13% to 5.48%. The stability of LYS228 in human plasma was demonstrated for at least 225 days at −75°C. This covers the longest storage period of plasma samples in this first-in-human study.

**(ii) Analysis of LYS228 in urine samples.** Concentrations of LYS228 in human urine were quantified via an established and validated analytical method; the urine samples were diluted and analyzed by reverse-phase liquid chromatography with tandem mass spectrometric detection. The linearity of the analytical method for analysis of LYS228 was validated (linear regression) in the range of 4 (lower limit of quantification) to 2,000 ng/ml using 50 μl of urine. The method was specific in human urine (maximum interferences, 13.08% of signal at LLOQ in blank plasma and 0.62% in zero sample). The interday accuracy and precision of the method were evaluated as the mean bias and precision of QC samples analyzed during 3 validation days: bias at LLOQ was 7.75%, and precision was 5.45%. Above the LLOQ, the biases were within the range of 6.25% to 11.83% and the precisions were within the range of 3.01% to 4.00%. The stability of LYS228 in human urine was demonstrated for at least 206 days at −75°C. This covers the longest period of storage of urine samples in this first-in-human study.

### Pharmacokinetic assessments.

Pharmacokinetic parameters were evaluated in all subjects at all dose levels. For the SAD group, blood samples were collected predose and at 1, 1.5, 2, 2.5, 3, 4, 6, 8, and 12 h after the start of the intravenous infusion. Urine was collected at baseline and during three time intervals: 0 to 4, 4 to 8, and 8 to 12 h after the start of the infusion. For the MAD, blood and urine were collected at similar time points or intervals as in the SAD, with the first dose on day 1 and on day 6. PK parameters, including *C*_max_, *T*_max_, AUC, *t*_1/2_, volume of distribution at steady state (*V*_ss_), and CL, were determined using the actual recorded sampling times and noncompartmental methods with Phoenix WinNonlin (version 6.4). Concentrations of LYS228 in urine were quantified via an established and validated LC-MS/MS method. The amount excreted (*A*_e_) for LYS228 was determined from the urine concentration and volume-time data. The renal clearance (CL_R_) was determined based on AUC and *A*_e_ available for the same time period. The linear trapezoidal rule was used for AUC calculation. Regression analysis of the terminal plasma elimination phase for the determination of *t*_1/2_ included at least three data points after *C*_max_. If the adjusted *R*^2^ value of the regression analysis of the terminal phase was less than 0.75, no values were reported for *t*_1/2_, AUC, or CL.

### Statistical methods.

Summary statistics were provided for PK parameters by treatment and sampling time point. PK endpoints (*C*_max_ and AUCs) for the SAD and MAD groups were assessed by fitting a dose proportionality model to the data following administration of single or multiple doses of LYS228 based on the following: PK parameter = α × dose^β^. The quality of the fit of the power model to assess dose proportionality was assessed by comparing the linear submodel against a saturated model with a different mean level at each concentration. In the case that there was evidence of lack of fit, alternative models were explored.
